# Bacterial Superantigens Promote Acute Nasopharyngeal Infection by *Streptococcus pyogenes* in a Human MHC Class II-Dependent Manner

**DOI:** 10.1371/journal.ppat.1004155

**Published:** 2014-05-29

**Authors:** Katherine J. Kasper, Joseph J. Zeppa, Adrienne T. Wakabayashi, Stacey X. Xu, Delfina M. Mazzuca, Ian Welch, Miren L. Baroja, Malak Kotb, Ewa Cairns, P. Patrick Cleary, S. M. Mansour Haeryfar, John K. McCormick

**Affiliations:** 1 Department of Microbiology and Immunology and the Centre for Human Immunology, Schulich School of Medicine & Dentistry, Western University, London, Ontario, Canada; 2 Department of Animal Care and Veterinary Services, Western University, London, Ontario, Canada; 3 Lawson Health Research Institute, London, Ontario, Canada; 4 Department of Basic Sciences, School of Medicine and Health Sciences, University of North Dakota, Grand Forks, North Dakota, United States of America; 5 Department of Medicine, Schulich School of Medicine & Dentistry, Western University, London, Ontario, Canada; 6 Department of Microbiology, University of Minnesota, Minneapolis, Minnesota, United States of America; National Institute of Allergy and Infectious Diseases, National Institutes of Health, United States of America

## Abstract

Establishing the genetic determinants of niche adaptation by microbial pathogens to specific hosts is important for the management and control of infectious disease. *Streptococcus pyogenes* is a globally prominent human-specific bacterial pathogen that secretes superantigens (SAgs) as ‘trademark’ virulence factors. SAgs function to force the activation of T lymphocytes through direct binding to lateral surfaces of T cell receptors and class II major histocompatibility complex (MHC-II) molecules. *S. pyogenes* invariably encodes multiple SAgs, often within putative mobile genetic elements, and although SAgs are documented virulence factors for diseases such as scarlet fever and the streptococcal toxic shock syndrome (STSS), how these exotoxins contribute to the fitness and evolution of *S. pyogenes* is unknown. Here we show that acute infection in the nasopharynx is dependent upon both bacterial SAgs and host MHC-II molecules. *S. pyogenes* was rapidly cleared from the nasal cavity of wild-type C57BL/6 (B6) mice, whereas infection was enhanced up to ∼10,000-fold in B6 mice that express human MHC-II. This phenotype required the SpeA superantigen, and vaccination with an MHC –II binding mutant toxoid of SpeA dramatically inhibited infection. Our findings indicate that streptococcal SAgs are critical for the establishment of nasopharyngeal infection, thus providing an explanation as to why *S. pyogenes* produces these potent toxins. This work also highlights that SAg redundancy exists to avoid host anti-SAg humoral immune responses and to potentially overcome host MHC-II polymorphisms.

## Author Summary

*Streptococcus pyogenes* is the most common cause of bacterial pharyngitis, also known as ‘strep throat’. However, this organism is also responsible for a range of other important human illnesses including necrotizing fasciitis and rheumatic heart disease (RHD). Indeed, complications from RHD and invasive infections by *S. pyogenes* are responsible for over one half million deaths per year in the world. *S. pyogenes* produces potent toxins called superantigens (SAgs), also known as the scarlet fever or erythrogenic toxins. SAgs have been studied for many years, yet we don't understand what purpose SAgs play in the life cycle of *S. pyogenes*. Rather than studying SAgs in the context of serious streptococcal disease, we studied the role of SAgs in a nasopharyngeal infection model. Our work demonstrates that for *S. pyogenes* to efficiently infect mice, the mice must express a human protein that is a receptor for the SAgs, and that *S. pyogenes* must produce SAgs. We further show that immunizing against SAgs prevents nasopharyngeal infection. This work demonstrates that SAgs are important factors for establishing infection by *S. pyogenes* and that SAgs may be potential candidates for inclusion within a *S. pyogenes* vaccine.

## Introduction

*S. pyogenes* (commonly known as the β-hemolytic Group A *Streptococcus*) is a prominent bacterial pathogen that causes a diverse range of clinical manifestations. Globally, *S. pyogenes* is responsible for over 600 million cases of pharyngitis, and more than one half million deaths primarily from complications of autoimmune rheumatic heart disease and invasive infections [1]. In addition, approximately 12% of school-aged children are asymptomatic carriers of this organism [2], and this ‘carriage’ state can last for years without the development of disease [3]. Although humans remain the only known natural reservoir for *S. pyogenes*, closely related streptococci such as *Streptococcus canis* and *Streptococcus dysgalactiae* lack host-specific adaptation. This indicates that the ancestor to *S. pyogenes* was unlikely to be human specific, and this further suggests that a primary feature in the evolution of *S. pyogenes* was the stringent adaptation to the human host [4]. Although morbidity associated with *S. pyogenes* is dependent on the ability to colonize and transmit within human populations, the molecular basis of the human specific tropism by *S. pyogenes* remains poorly understood.

One group of ‘trademark’ virulence factors produced by *S. pyogenes* are the bacterial superantigens (SAgs), also commonly referred to as the erythrogenic toxins or the streptococcal pyrogenic exotoxins [5]. There are at least 14 genetically distinct streptococcal SAgs [6] often encoded within mobile, or putatively mobile, genetic elements [7]–[9]. Thus, different *S. pyogenes* strains typically encode for distinct repertoires of multiple SAgs. These toxins function by engaging lateral surfaces of both MHC class II (MHC-II) molecules and T cell receptor (TCR) β-chains [10]; these unconventional interactions can force the activation of enormous numbers of T cells. Indeed, human MHC-II molecules are known host factors fundamental to the development of severe streptococcal disease [11]–[14], and the ability of MHC-II to modulate severity of invasive streptococcal disease has been linked directly to SAgs [12], [15]. Although SAgs are well recognized in the pathogenesis of scarlet fever [16], [17] and the streptococcal toxic shock syndrome (TSS) [18], [19], in what context these toxins contribute to the fitness and life cycle of *S. pyogenes* is unknown. Thus, since a major biological niche for *S. pyogenes* is the upper respiratory tract, we hypothesized that SAgs have likely evolved to function in the context of asymptomatic nasopharyngeal colonization and/or pharyngitis, rather than in the context of severe invasive disease.

Here we show that mice expressing human MHC-II molecules are highly susceptible to acute nasopharyngeal infection by *S. pyogenes*. Furthermore, we demonstrate that *S. pyogenes* MGAS8232 requires the streptococcal pyrogenic exotoxin A (SpeA) SAg to cause acute nasopharyngeal infection. In addition, immunization with toxoid SpeA is protective for nasopharyngeal infection by wild-type *S. pyogenes* MGAS8232. This work indicates that the streptococcal SAgs play an important role in the life cycle of *S. pyogenes* by promoting the initial stages of infection, and that these toxins should be further considered as potential vaccine targets to prevent *S. pyogenes* nasopharyngeal carriage.

## Results

### Human Leukocyte Antigen (HLA) molecules are critical host factors for nasal infection by *S. pyogenes*

**Figure 1:**
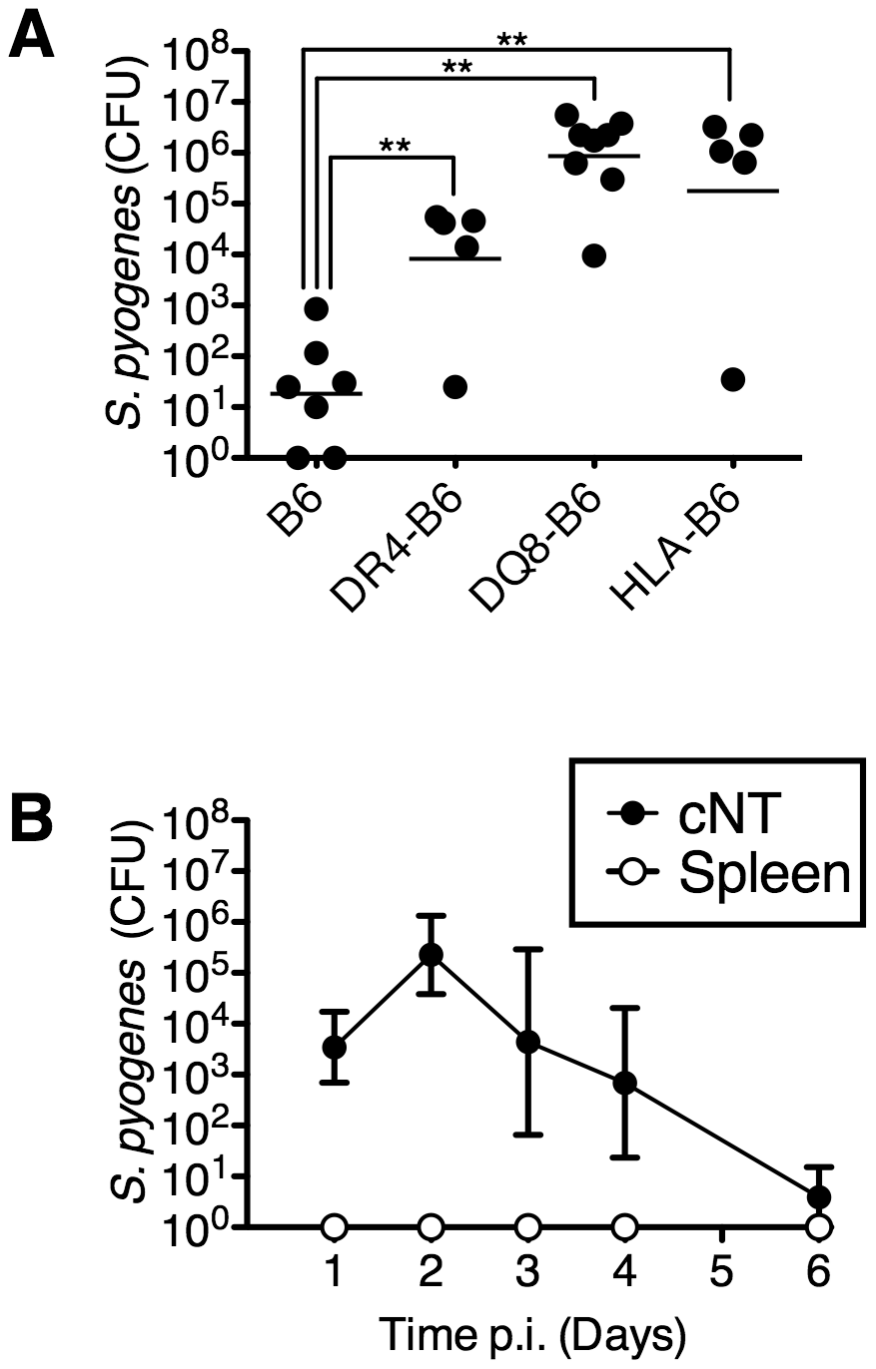
MHC-II is an important determinant of *S. pyogenes* nasopharyngeal infection in mice. (A) Individual mice were nasally inoculated with ∼1×10^8^ bacterial colony forming units (CFU) with MGAS8232 and nasopharyngeal CFUs were assessed at 48 h. Each symbol represents CFUs from an individual mouse. (B) Time-course analysis of MGAS8232 inoculated HLA-B6 mice. CFUs were determined from the cNTs and spleens (n≥3 per group) at the indicated time points. Statistical significance is displayed as **p<0.01 by Student's *t*-test.

*S. pyogenes* infection of the nasopharynx in mice is a model for pharyngeal infection in humans [20], [21]. We first evaluated the influence of human MHC-II on mouse nasopharyngeal infection by *S. pyogenes*. Nasal inoculation of C57BL/6 (B6) mice with ∼1×10^8^ CFU of *S. pyogenes* MGAS8232 resulted in very low bacterial recovery from the nasal mucosa [herein referred to as the complete nasal turbinates (cNT)] at 48 h (**Figure 1A**). However, ‘humanized’ B6 mice that express HLA-DR4 (DR4-B6 mice) contained ∼100-fold more CFUs of *S. pyogenes* compared to wild-type B6 mice, and mice that expressed HLA-DQ8 (DQ8-B6 mice), or both HLA-DR4 and HLA-DQ8 (herein referred to as HLA-B6 mice), contained ∼10,000-fold more CFUs than wild-type B6 mice (**Figure 1A**). In a time course analysis of this acute infection model, ∼5×10^3^ bacterial CFUs of *S. pyogenes* MGAS8232 were recovered at 24 h, which increased by ∼100-fold at 48 h, and were subsequently cleared after one week (**Figure 1B**). Importantly, MGAS8232 did not become invasive as viable *S. pyogenes* cells were not recovered from the spleen (**Figure 1B**) or blood (data not shown) in HLA-B6 mice at any time point. These data reveal that human MHC-II molecules are important host factors for acute nasopharyngeal infection by *S. pyogenes*.

### HLA-B6 mice are responsive to SpeA and SmeZ

**Figure 2:**
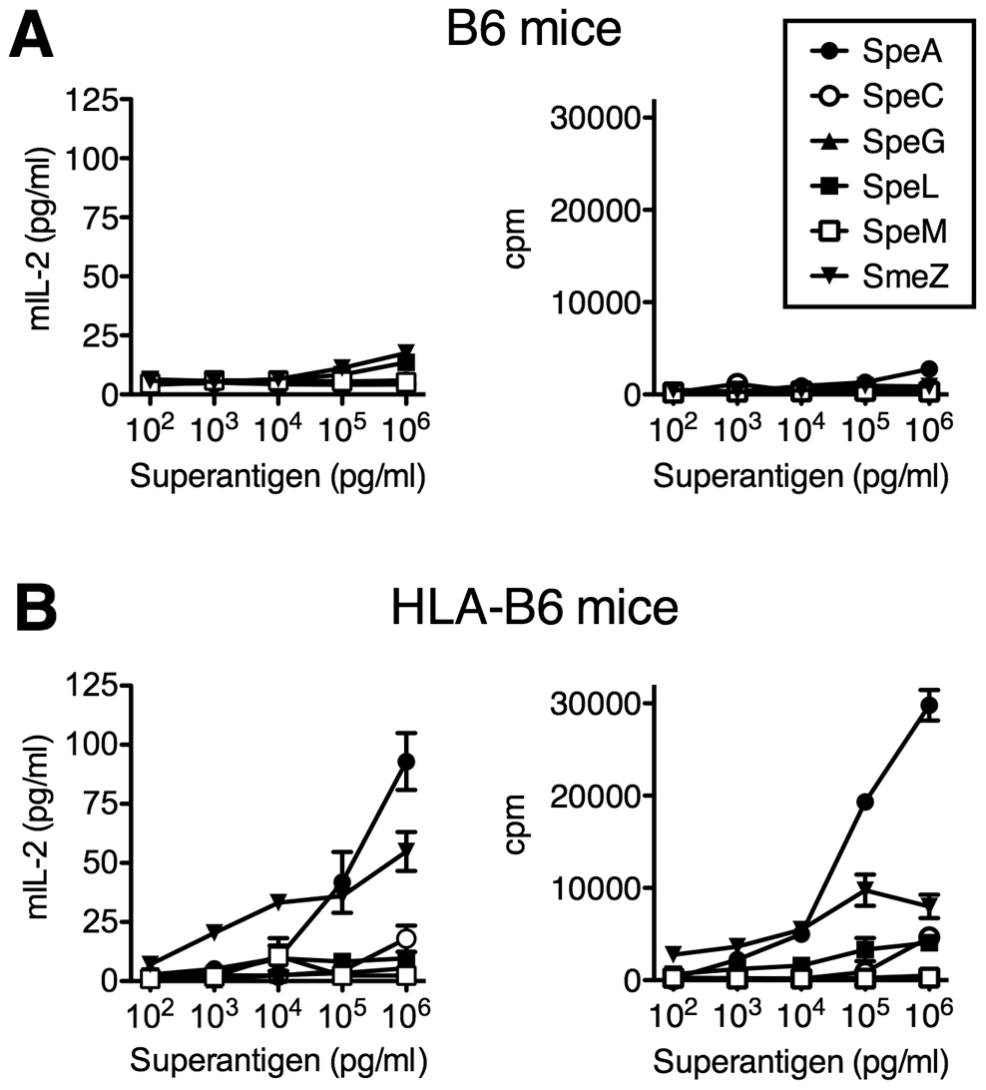
HLA-B6 mice are responsive to SpeA and SmeZ. Splenocytes (2×10^5^/well) from (A) B6 or (B) HLA-B6 mice were treated with serial 10-fold titrations of indicated SAgs. Mouse IL-2 (mIL-2) production was assessed at 18 h, and proliferation, expressed as count per minute (cpm), was assessed after 72 hours. Data shown is from a representative experiment completed in triplicate.

To investigate the potential role of SAgs for nasopharyngeal infection in HLA-mice, we mined the genome of *S. pyogenes* MGAS8232 and confirmed that this strain encodes for six genetically distinct SAgs (SpeA, SpeC, SpeG, SpeL, SpeM and SmeZ) [22]. The mature coding region for each SAg gene was cloned, recombinant SAgs (rSAg) were expressed and purified, and each was shown to activate human T cells (data not shown). To assess activity of the rSAgs in HLA-mice, *ex vivo* splenocyte activation was evaluated. B6 splenocytes demonstrated little capacity for activation by all six rSAgs as measured by mouse IL-2 production and proliferative responses (**Figure 2A**). However, splenocytes from HLA-B6 mice showed enhanced responses to both the SpeA and SmeZ rSAgs in a dose-dependent manner (**Figure 2B**). These data indicate that both SpeA and SmeZ function in HLA-B6 mice and suggest that SAgs encoded within *S. pyogenes* MGAS8232 may contribute to the ability of *S. pyogenes* to infect the nasopharynx of HLA-mice.

### Construction of superantigen deficient *S. pyogenes*

**Figure 3:**
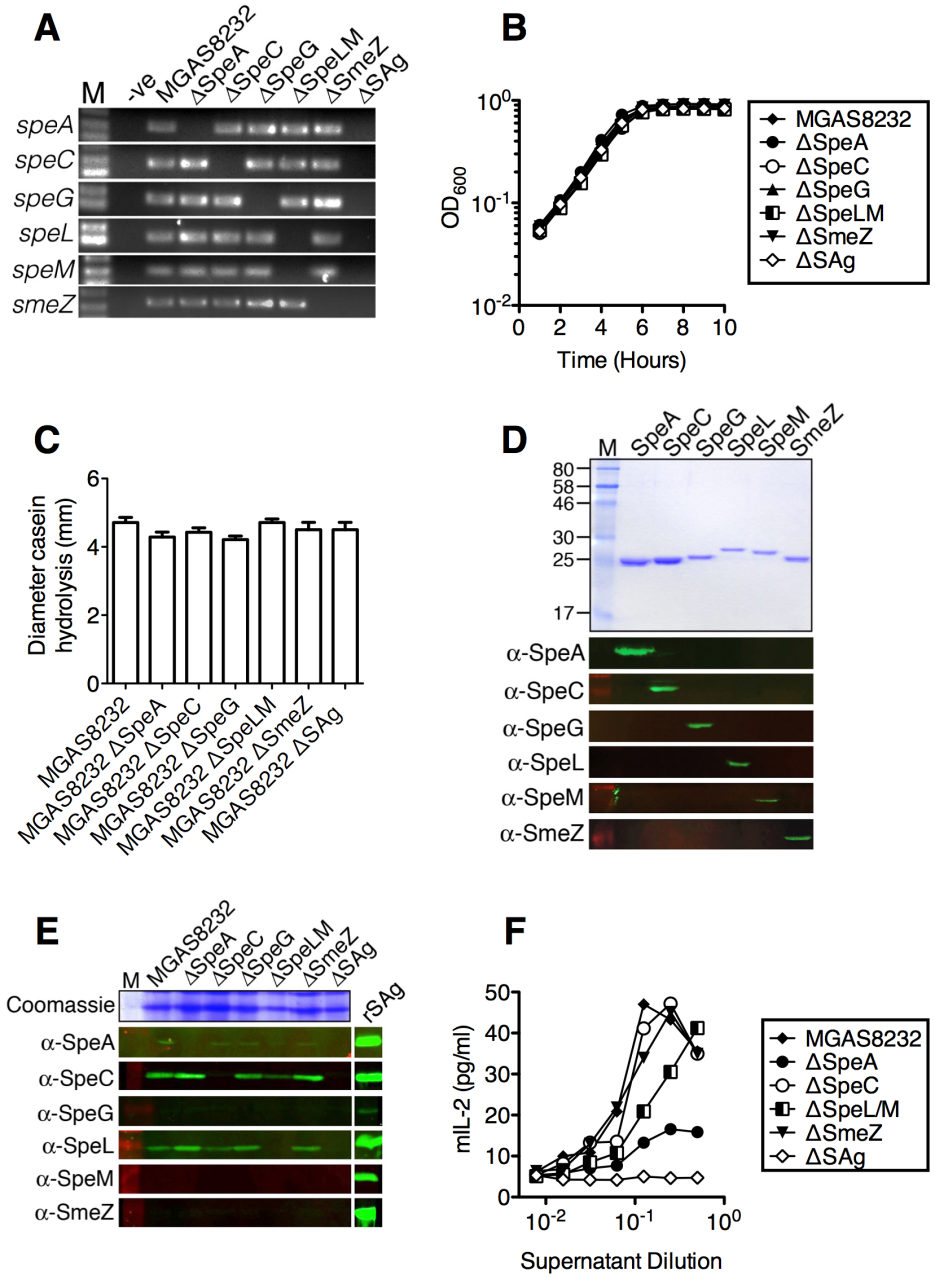
Generation of isogenic *S. pyogenes* genetic mutants lacking superantigen genes. Individual SAg genes were systemically deleted in *S. pyogenes* MGAS8232 as detailed in Materials and Methods. (A) Genetic SAg deletions were confirmed by PCR with primers internal to each gene. Growth curves (B) and protease activity (C) of the indicated mutants. (D) Recombinant SAgs (SDS-PAGE; top panel) and Western blot experiments (bottom six panels) to determine specificity of rabbit polyclonal antisera to distinct recombinant SAg proteins. (E) Streptococcal SAg deletion strains were assessed for production of each SAg (100-fold concentrated supernatants) by Western blotting. (F) Activation potential using mouse IL-2 (mIL-2) assays using supernatant titrations to stimulate splenocytes (2×10^5^/well) from HLA-B6 mice.

In order to determine empirically if SAgs were direct contributors to the enhanced nasopharyngeal infection phenotype in HLA-mice, a series of precise, in-frame deletions were generated within the coding regions for each SAg gene in *S. pyogenes* MGAS8232 (**Figure 3A**). As *speL* and *speM* are encoded in tandem, these SAgs were deleted together. In addition, we generated a complete SAg deletion strain lacking the coding regions for all six SAg genes (MGAS8232 ΔSAg) (**Figure 3A**). Each of the *S. pyogenes* deletion strains grew comparably to wild-type MGAS8232 *in vitro* (**Figure 3B**). Furthermore, the various mutants did not show any alterations in protease (SpeB) activity (**Figure 3C**). To evaluate SAg production in these strains *in vitro*, we generated rabbit polyclonal antibodies to each rSAg. The specific rabbit antisera recognized each of the expected SAgs without cross-reacting with others (**Figure 3D**). Next, we assessed *in vitro* production of the SAg proteins for the wild-type and isogenic SAg deletion strains. The production of both SpeC and SpeL from wild-type *S. pyogenes* MGAS8232 was clearly detectable by Western blot analysis, while SpeA was weakly detected (**Figure 3E**). Importantly, for each of these three SAgs, the toxin was not made by the appropriate deletion mutant. To evaluate SAg activity from the different *S. pyogenes* mutants, we tested the supernatants from the isogenic *S. pyogenes* strains for the ability to activate HLA-B6 mice splenocytes. Consistent with the Western blot analysis (**Figure 3E**), and the ability of select SAgs to activate these cells (**Figure 2B**), the largest reduction in activity for the individual deletion strains was for MGAS8232 ΔSpeA, whereas the MGAS8232 ΔSAg mutant did not activate splenocytes above background levels (**Figure 3F**).

### The SpeA superantigen is required for *S. pyogenes* MGAS8232 acute nasopharyngeal infection in HLA-mice

**Figure 4:**
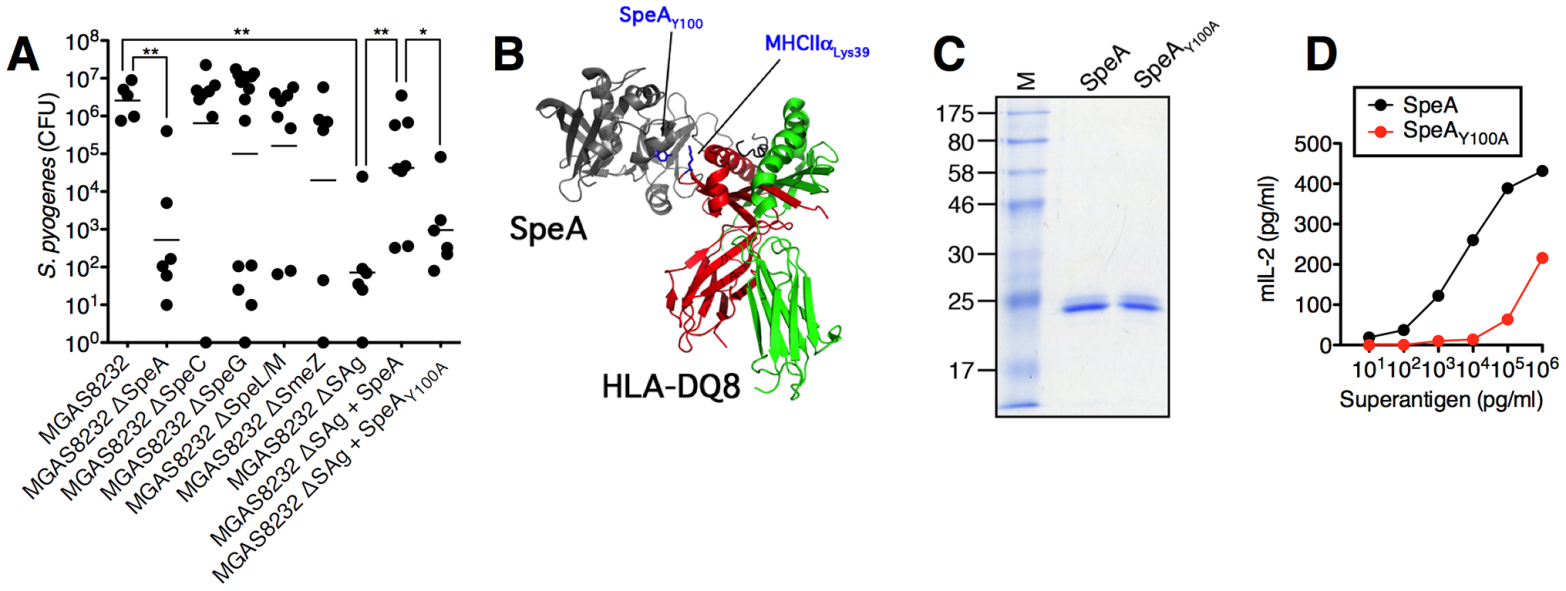
Superantigens are critical for *S. pyogenes* nasopharyngeal infection in mice. (A) Individual HLA-B6 mice were nasally inoculated with the indicated MGAS8232 strains [∼1×10^8^ (range 0.6–1.4×10^8^) CFUs, and nasopharyngeal CFUs were assessed at 48 h. Each symbol represents an individual mouse. (B) Ribbon diagram model of SpeA in complex with human MHC II with relevant amino acids highlighted. (C) Recombinant SpeA proteins (2 µg). (D) Dose response of SpeA and SpeA_Y100A_ for activation of HLA-B6 splenocytes (1×10^6^/well). Mouse IL-2 (mIL-2) production was assessed at 18 h. Statistical significance is displayed as *p<0.05 or **p<0.01 by Student's t test.

We next evaluated all of the isogenic *S. pyogenes* MGAS8232 strains for the ability to infect HLA-B6 mice. MGAS8232 ΔSpeA demonstrated a striking reduction in bacterial CFUs recovered at 48 h compared to wild-type MGAS8232 (**Figure 4A**). Although some variability was seen for the other mutants *in vivo* (ΔSpeC, ΔSpeG, ΔSpeLM and ΔSmeZ), multiple animal experiments demonstrated that each was capable of infecting the HLA-mice to wild-type levels. MGAS8232 ΔSAg however, resembled wild-type MGAS8232 recovery in B6 mice (**Figures 1A** **and** **4A**). In order to genetically complement the MGAS8232 ΔSAg strain, and to determine if a single SAg could promote infection, we introduced the wild-type *speA* gene (and the corresponding *speA* promoter) into the chromosome of MGAS8232 ΔSAg between the *pepO* and *tsf* genes. The wild-type SpeA complemented strain enhanced infection significantly above MGAS8232 ΔSAg that was not statistically different from wild-type MGAS8232 (**Figure 4A**). To assess the requirement for SAg activity for the infection phenotype, we constructed a SpeA MHC-II binding mutant based on a structural model of SpeA in complex with HLA-DQ8 (**Figure 4B**). This model predicted SpeA Tyr^100^ would interact with the conserved MHC-II α-chain Lys^39^ and both of these equivalent residues in the staphylococcal enterotoxins, and HLA-DR1, respectively, have been shown to be important for SAg function [23], [24]. Recombinant SpeA_Y100A_ (**Figure 4C**) demonstrated ∼100-fold reduction in potency relative to wild-type SpeA (**Figure 4D**) and thus we introduced *speA_Y100A_* into MGAS8232 ΔSAg in a similar fashion to the wild-type *speA* complementation experiment. The *speA_Y100A_*-complemented strain was significantly reduced compared to the wild-type SpeA complemented stain, and although CFUs were not statistically increased over MGAS8232 ΔSAg, there was a trend for enhanced infection by ∼1 log. We believe this potential increase may reflect the residual activity present in the SpeA_Y100A_ mutant (**Figure 4A**). Furthermore, using qRT-PCR we confirmed that the *in vitro* expression of *speA_Y100A_* was similar to *speA_WT_* between the two complemented strains (data not shown). These collective data indicate that the enhanced nasopharyngeal infection by *S. pyogenes* MGAS8232 in HLA-B6 mice is due to the production of the SpeA exotoxin, and that SAg function is required.

### *S. pyogenes* infects the nasal turbinates and induces superantigen-dependent inflammation

**Figure 5:**
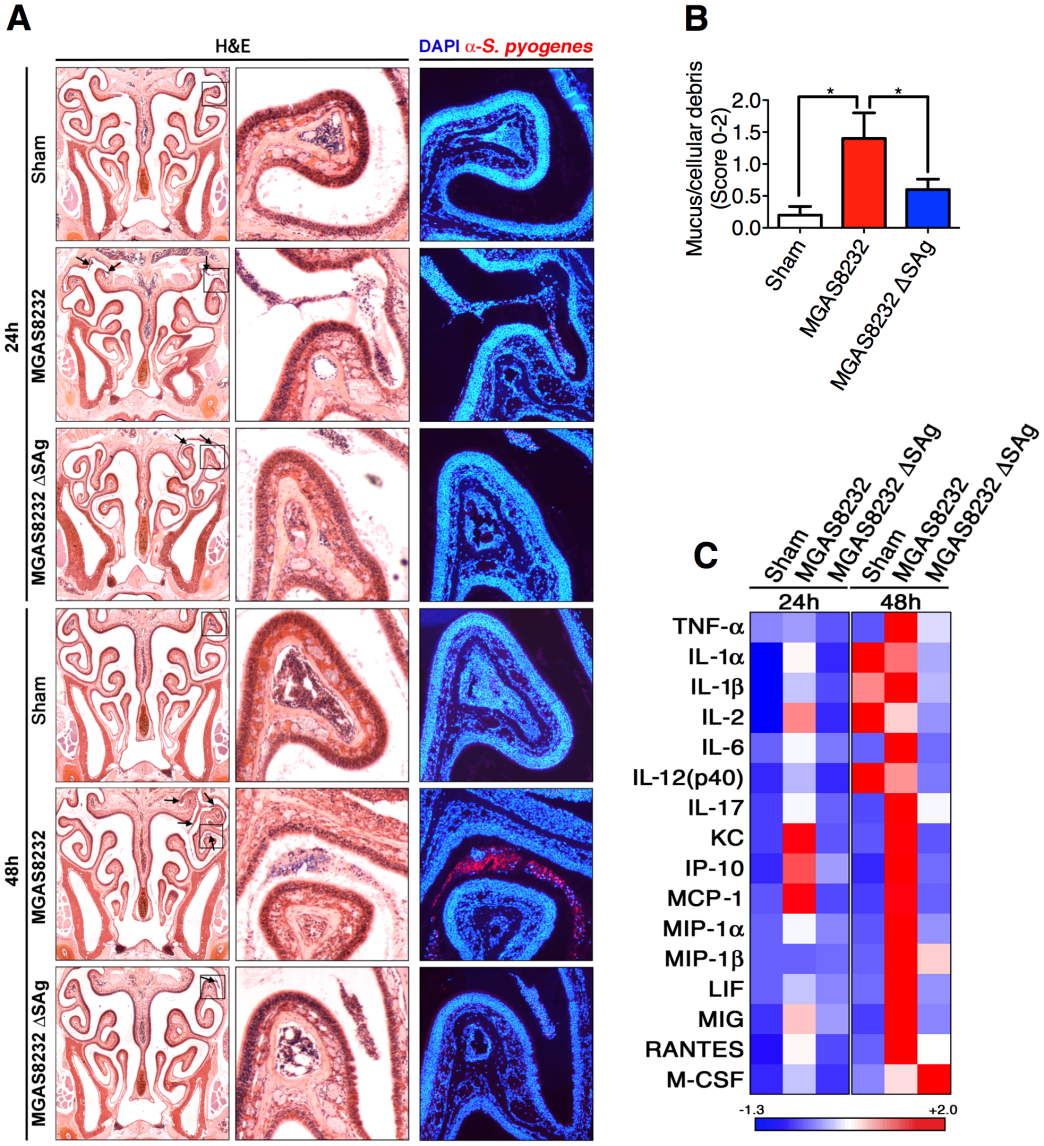
Nasopharyngeal infection of HLA-B6 mice with wild-type *S. pyogenes* MGAS8232 localizes to the nasal turbinates and induces a SAg-dependent inflammatory response. (A) Coronal 5 µm sections of mouse heads stained with H&E stain or with DAPI (blue) and α-*S. pyogenes* (red) antibody. Shown are representative sections at 24 h and 48 h time points for sham treated, wild-type *S. pyogenes* MGAS8232 and MGAS8232 ΔSAg strains. Right panels are close up views from the boxed sections. Arrows indicate inflammatory cells containing *S. pyogenes*. (B) Histological assessment of nasal tissue sections from *S. pyogenes* MGAS8232 and MGAS8232 ΔSAg infected HLA-mice. Differences in pathological severity were assessed by examination of H&E stained sections at 48 h. Sections (n = 5 per group) were scored (0–2) for the presence and severity of mucus, red blood cells, and nucleated cellular debris on the surface of the respiratory epithelium. Statistical significance is displayed as *p<0.05 by Student's t test. (C) Heat map of multiplex cytokine arrays from treatment groups as in Panel A (n≥3 mice per group). Data shown represent the mean cytokine responses that displayed significant differences within each time group. Values for each row were normalized to have a mean of zero and a variance of one. Corresponding quantitative data and statistical analyses are shown in Figure S2.

To gain insight into the *S. pyogenes* infection process in HLA-B6 mice, we generated coronal sections of the nasal passage for sham, wild-type MGAS8232, or MGAS8232 ΔSAg treated mice, at both 24 and 48 h. Sections were stained with H&E, as well as DAPI (blue) and an anti-*S. pyogenes* fluorescent antibody (red). As predicted, there was no evidence of *S. pyogenes* infection seen in the sham treated mice at 24 or 48 h (**Figure 5A**). Both wild-type MGAS8232 and MGAS8232 ΔSAg demonstrated sparse, but detectable *S. pyogenes* cells, within the upper nasal turbinates at 24 h (**Figure 5A**). This was consistent with bacterial counts from MGAS8232 ΔSAg infected mice at 24 h (975±475 CFU/cNT; n = 3 mice) that did not differ significantly from wild-type MGAS8232 infected mice (**Figure 1B**). Consistent with the CFU data (**Figure 4A**), very few MGAS8232 ΔSAg cells were detected microscopically at 48 h, whereas wild-type MGAS8232 produced a robust infection that was localized to the upper nasal turbinates (**Figure 5A**; 48 h MGAS8232 boxed insets). H&E-stained sections from 48 h post-infection (n = 5 mice per group, 2 sections per mouse) were scored in a blinded fashion for the presence and severity of mucus, red blood cells, and nucleated cellular debris on the surface of the respiratory epithelium and neuroepithelium. This analysis revealed no significant findings for the sham treated mice, and mice infected with MGAS8232 ΔSAg displayed only mild neutrophilic infiltration in the sub-epithelial spaces, and mild epithelial disruption (**Figure 5B**). However, mice infected with wild-type MGAS8232 demonstrated significant signs of inflammation including sloughed cellular debris into the nasal cavity lumen, and marked neutrophilic infiltration and epithelial cell disruption with evidence of cocci on the epithelial surface, and evidence of hypersecretory activity from the neuroepithelium (**Figure 5B**). Flow cytometric analysis of total cNT preparations from mice treated with the 3 groups (n = 4 mice per group) however, revealed few overall changes in immune cell percentages, although a significant decrease in the dendritic cell (DC) (CD11c^+^) population was revealed in the wild-type MGAS8232 infected animals compared with MGAS8232 ΔSAg infect mice, and the analysis also showed a trend for increased neutrophils (GR1^+^) in wild-type MGAS8232 infected animals (**Figure S1**) that was consistent with the histological analyses. We did not observe any changes in immune cell populations in the spleen or lymph nodes (**Figure S1**). However, cytokine analysis of homogenized cNTs demonstrated an early inflammatory type or T_H_1-skewed response of the wild-type MGAS8232 infected mice compared with the MGAS8232 ΔSAg strain, including enhanced production of IL1α, IL-2, IL-6 and IL-17, as well as the chemokines KC, IP-10 and MCP-1 (**Figures 5C and S** **2**). By 48 h, the wild-type MGAS8232 infected mice displayed a robust chemokine response consistent with the high numbers of bacterial cells by this time point. Taken together, these data are consistent with an early SAg-dependent inflammatory environment within the nasal turbinates where *S. pyogenes* could both survive and rapidly expand to high numbers over the initial 48 h of infection.

### Superantigen-neutralizing antibodies inhibit nasopharyngeal infection by *S. pyogenes* in HLA-B6 mice

**Figure 6:**
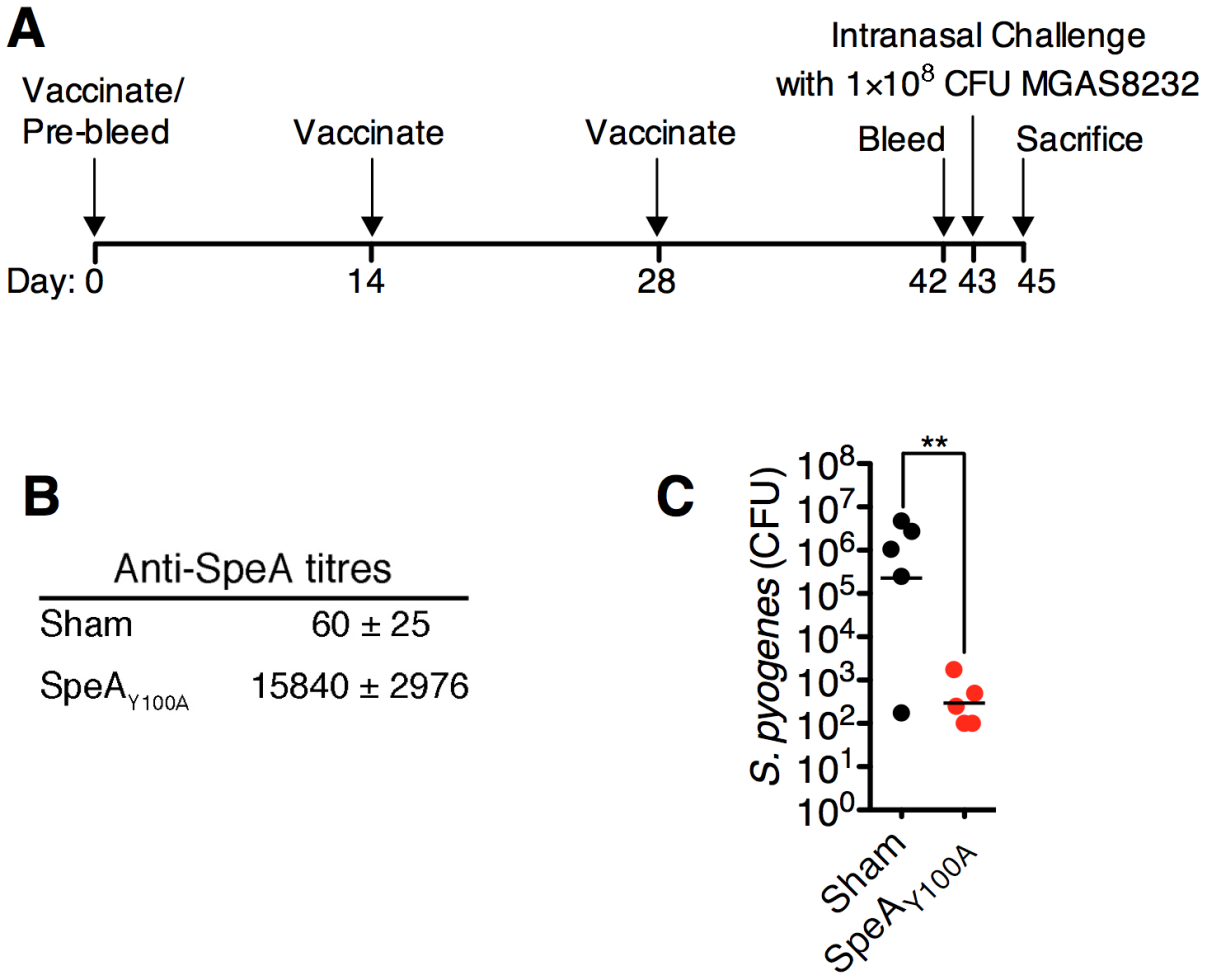
Vaccination with toxoid SAg inhibits *S. pyogenes* nasopharyngeal infection in mice. (A) Time-line of vaccination protocol. (B) Anti-SpeA IgG antibody titres (mean ± SEM) from HLA-B6 mice (n = 5 mice per group), either sham vaccinated, or vaccinated with SpeA_Y100A_ as described in Materials and Methods. (C) Sham or SpeA_Y100A_ vaccinated HLA-B6 mice were nasally inoculated with ∼1×10^8^ bacterial CFUs of *S. pyogenes* MGAS8232. Nasopharyngeal CFUs were assessed at 48 h. Each symbol represents an individual mouse. Statistical significance is displayed as **p<0.01 by Student's *t*-test.

Given the prominent nasopharyngeal infection phenotype for *S. pyogenes* MGAS8232 that was SpeA-dependent (**Figures 4A** **and** **5A**), we tested the ability of humoral immunity to SpeA to influence nasopharyngeal infection by *S. pyogenes* MGAS8232 (**Figure 6A**). We vaccinated HLA-mice with the SpeA_Y100A_ toxoid and these mice developed high anti-SpeA IgG titres compared with sham-vaccinated mice (**Figure 6B**). Following nasal inoculation with ∼1×10^8^ CFU of wild-type MGAS8232, SpeA_Y100A_-vaccinated mice showed a dramatic reduction in infection (**Figure 6C**) that was similar to the MGAS8232 ΔSpeA mutant (**Figure 4A**), while sham-vaccinated mice were infected efficiently (**Figure 6C**). These data indicate that humoral immunity to appropriate SAgs can inhibit nasopharyngeal infection in SAg-sensitive mice.

## Discussion

Bacterial SAgs from *S. pyogenes* are well recognized as important virulence factors for the development of serious toxin-mediated diseases such as the streptococcal TSS [5]. However, given that streptococcal TSS is rare, and since the disease has a very high mortality rate (∼45%) [25], it is unlikely that the induction of streptococcal TSS provides an evolutionary advantage for *S. pyogenes*. Thus, the actual biological function of these toxins has remained an enigma. We reasoned that since a major niche for *S. pyogenes* is the epithelial surfaces of the nasopharynx, we sought to evaluate the influence of SAgs in this context. Herein, we provide important evidence that acute infection of the upper respiratory tract by *S. pyogenes* MGAS8232 is enhanced dramatically in mice expressing human MHC-II, and that this phenotype is directly attributed to SpeA. A logical interpretation of these findings is that the true biological function of SAgs, in terms of the life cycle of *S. pyogenes*, is to promote the initial establishment of infection in humans.

This work also provides further evidence that host MHC-II molecules are a central component of the human specific tropism of *S. pyogenes*. Human MHC-II [11]–[14], human plasminogen [26], [27], and human CD46 [28] are each known to contribute to the development of invasive streptococcal disease, although the influence of these latter host factors have not yet been tested in a nasopharyngeal infection model. *S. pyogenes* produces a hyaluronic acid capsular polysaccharide [29] that is important for nasopharyngeal colonization in mice [30], and the hyaluronic acid binding receptor CD44 is an important receptor for *S. pyogenes* [31]. However, the conserved amino acid sequences, structures, and binding affinities of human and mouse CD44 for hyaluronan [32] would argue against a specific role of CD44 in the human-specific tropism of *S. pyogenes*.

Although there were no significant differences in CFUs at 24 h comparing wild-type *S. pyogenes* and the SAg-deficient strain, wild-type MGAS8232 was able to expand rapidly between 24 h to 48 h by ∼2 logs (**Figure 1B**), which was entirely consistent with the immunofluorescence staining of the nasal passages (**Figure 5A**). Although there were few differences in immune cell percentages from the cNT by 48 h, other than a clear decrease in CD11c^+^ leukocytes for wild-type MGAS8232 infected mice (**Figure S1**), *S. pyogenes* did induce a SAg-dependent Th1/inflammatory cytokine response, which was accompanied by the influx of innate immune cells by 48 h (**Figure 5A**). Selective depletion of CD11c^+^ DCs in mice resulted in enhanced dissemination of *S. pyogenes* from a subcutaneous infection into lymph nodes and the liver, demonstrating that DCs are contributors to host defence against *S. pyogenes* [33]. However, some strains of S. *pyogenes* can also induce DC apoptosis in a streptolysin O-dependent manner [34], which may have contributed to the decrease in CD11c^+^ populations. Nevertheless, we favour the hypothesis that *S. pyogenes* has provoked a localized and SAg-mediated cytokine response that resulted in a state of transient immunosuppression allowing *S. pyogenes* to escape myeloid cell mediated killing. In support of this hypothesis, staphylococcal enterotoxin B has been demonstrated *in vivo* to induce a transient (∼48 h) Vβ-unrestricted immunosuppressive response in T cells with the inability to produce IL-2, that also caused decreased numbers of splenic CD11c^+^ dendritic cells [35]. These responses were very consistent with mouse cytokine responses (**Figure 5C**) and immune cell analyses (**Figure S1**) of MGAS8232 infected HLA-B6 mice. By 48 h, however, a robust IL-6 and IL-17 response was produced (**Figures 5C and S** **2**), concurrent with the high numbers of *S. pyogenes*, and each of these cytokines are known to be important for *S. pyogenes* control [21], [36]. Alternatively, inflammation induced epithelial cell damage within the nasal turbinates (**Figure 5B**) may have promoted access to host cell adhesive factors to allow for the initial establishment of infection. The very weak cytokine response to the MGAS8232 ΔSAg strain was somewhat surprising, yet this finding may support this second hypothesis where in the absence of functional SAg, *S. pyogenes* are rapidly cleared through primarily mucociliary clearance mechanisms. Although *S. pyogenes* is capable of internalization into epithelial cells, the evidence indicates that *S. pyogenes* does not replicate efficiently within epithelial cells [37], [38], and thus we do not predict a role for enhanced intracellular survival based on SAg expression.

SAgs have also been studied directly in the context of live invasive streptococcal disease using defined genetic knockout strains. Earlier work utilizing a *S. pyogenes* myositis model in HLA-DQ8 transgenic mice demonstrated clear Vβ-specific alterations during infection, as well as SpeA-dependent conjunctivitis, hyperplasia of the lymph nodes and spleen, and T cell infiltration into the liver, yet the SpeA knockout strain demonstrated no difference in overall mortality compared with the wild-type counterpart [14]. Additionally, although SmeZ is dominant in the *speA*- and *speC*-negative M89 isolate *S. pyogenes* H293, genetic disruption of *smeZ* did not alter bacterial clearance or mortality in a peritoneal infection model [39]. Thus, a picture has emerged where individual SAgs may not contribute to *S. pyogenes* survival during invasive disease. Nevertheless, streptococcal SAgs can cause TSS directly in experimental animal models [40]–[42], human MHC-II molecules contribute to mouse mortality during invasive infections [12], [14], and in severe invasive human infections, streptococcal TSS is a major contributor to overall mortality [25].

In the nasal infection model presented here, despite the high number of bacterial cells recovered from wild-type *S. pyogenes* infected HLA-B6 mice at 48 h, *S. pyogenes* did not become invasive (**Figure 1B**), and the infection was reduced to very low levels after about 7 days. In humans, symptomatic pharyngitis infections typically last for about one week without antibiotic treatment [43], and thus the presented model appears to be a reasonable approximation of acute upper respiratory tract infection in humans. However, whether the model could replicate a longer term asymptomatic colonization state is unlikely, as many patients can harbour *S. pyogenes* for extended time periods, potentially lasting over 2 years [3].

Although our data demonstrate that SpeA is critical for the infection phenotype in HLA-B6 mice, the genome of *S. pyogenes* MGAS8232 also encodes for 5 other SAgs, each which is known to activate human T cells [44]–[47]. Although SpeC was the predominant SAg secreted from MGAS8232 *in vitro* (**Figure 3E**), the lack of a phenotype for the MGAS8232 ΔSpeC mutant was somewhat predicted, as this SAg does not activate murine T cells [48] (**Figure 2**). Although recombinant SmeZ did potently activate splenocytes from HLA-B6 mice, and SmeZ is the primary immunoactive SAg for some *S. pyogenes* strains [39], we did not detect expression of SmeZ *in vitro* from wild-type MGAS8232 (**Figure 3E**), which likely contributed to the inability of SmeZ to compensate functionally in the MGAS8232 ΔSpeA strain. However, the MGAS8232 ΔSAg strain complemented with wild-type SpeA did not appear to fully restore the infection phenotype, and the lack of SmeZ production could potentially be responsible for this result. The remaining SAgs showed very weak activity for the activation of splenocytes from HLA-mice and thus they do not play an important role for infection in this model.

The vaccination experiments provide further evidence, independent of the genetic deletion strains, of the critical role played by SpeA during the infection. Neutralization of SAg activity by antibodies is consistent with clinical evidence that has established a link between the lack of streptococcal SAg-neutralizing antibodies and the development of streptococcal TSS [49]-[51]. Also, neutralizing antibodies are known to protect against experimental STSS in rabbits [40], [41]. Thus, pre-existing anti-SAg antibodies may potentially inhibit infection by specific strains of *S. pyogenes*, yet *S. pyogenes* could theoretically circumvent this through up-regulation of additional SAgs for which neutralizing antibodies are absent. Since it is clear that different strains of *S. pyogenes* encode different repertoires of SAgs [7], *S. pyogenes* may also potentially alter SAg expression patterns to engage different host MHC-II molecules.

The SAgs remain well-recognized virulence factors for *S. pyogenes*. However, this work demonstrates that their genuine contribution to the life cycle of this pathogen is likely to promote the establishment of pharyngitis, or potentially asymptomatic colonization, in genetically susceptible individuals expressing SAg-responsive MHC-II molecules. The redundancy of SAgs within *S. pyogenes* has also remained unexplained, and this work further illustrates that this may exist, in part, to overcome the highly polymorphic nature of human MHC-II molecules, and also to avoid natural host immunity to SAgs. Thus, SAgs are important contributors to the complex genotype-phenotype relationship that exists between *S. pyogenes* and humans, and these toxins should be considered further as valid targets for vaccination studies to impede the enormous burden of disease by this versatile pathogen.

## Materials and Methods

### Ethics statement

All animal experiments were in accordance with the Canadian Council on Animal Care Guide to the Care and Use of Experimental Animals. The animal protocol (#2009-038) was approved by the Animal Use Subcommittee at Western University.

### Bacteria

*S. pyogenes* MGAS8232 is an M18 serotype that was isolated from a patient with acute rheumatic fever in Utah in 1987 and the genome has been sequenced and fully annotated [22]. *S. pyogenes* strains were grown in Todd Hewitt media supplemented with 1% (w/v) yeast extract. Deletions were made for all of the SAg genes using the pG^+^host5 system [52], [53]. All recombinant plasmids were built with standard molecular procedures using *Escherichia coli* XL1-blue as the cloning host [54]. Briefly, deletion constructs were generated by amplification of ∼500 bp of DNA on either side of the relevant SAg gene (Primers are listed in **Table S1**) and cloned into pG^+^host5. Flanking DNA included the first and last 8 codons for each SAg gene to generate precise, markerless and in frame deletions of each SAg gene. Plasmids were electroporated (Bio-Rad Gene Pulser XCell) into *S. pyogenes* MGAS8232 and single crossover integrations were selected at 40°C under erythromycin (1 µg ml^−1^) selection. PCR confirmed single crossover integrations were subcultured without antibiotics at 30°C and single clones were screened for a loss of erythromycin resistance, and double crossover gene disruptions were confirmed by PCR. In each case, appropriate PCR products were sequenced to confirm the expected deletion. All mutants were confirmed to lack growth alterations using Bioscreen C (Piscataway, NJ, USA) assays. To assess for protease activity, *S. pyogenes* strains were grown on dialyzed brain heart infusion agar containing 1.5% skim milk. Five microliters of OD_600_ 0.1 *S. pyogenes* mutants were inoculated into 2 mm holes punched in the plates and incubated at 37°C for 24 hours. The ability of each strain to hydrolyze casein was assessed by the diameter of the zones of clearing. For *speA* and *speA_Y100A_* complementation experiments, wild-type and mutant *speA* from MGAS8232 (including the native promoter) were individually ‘knocked in’ using the pG^+^host5 system to the non-coding region between genes encoding endopeptidase O (*pepO*) and elongation factor-Ts (*tsf*) and confirmed by PCR and DNA sequencing.

### Superantigens and antibodies

Genes encoding for SpeA, SpeG, SpeL, SpeM, and SmeZ, lacking nucleotides encoding the predicted signal peptides, were PCR amplified and cloned into a modified pET-28a vector to introduce an engineered tobacco etch virus (TEV) protease cleavage site downstream from a His_6_ tag. Cloning of SpeC into the pET-41a vector has been described [55], and SmeZ was cloned in a similar manner to SpeC. All recombinant SAgs were produced by 200 µM isopropyl β-D-1-thiogalactopyranoside (IPTG)-induced expression in *E. coli* BL21(DE3), purified via nickel affinity chelation chromatography, and His_6_ tags were removed using autoinactivation resistant His_6_::TEV protease [56], as described [57], [58]. Proteins were run on 12% separating SDS-PAGE gels and Western blots were visualized on a LI-COR Odyssey (LI-COR Biosciences) using IRDye800 conjugated donkey anti-rabbit IgG as the secondary antibody (Rockland Inc.). All recombinant SAgs ran as discrete homogenous bands by SDS-PAGE (**Figure 3D**). Purified and lyophilized SpeA, SpeC, SpeG, SpeL, SpeM, and SmeZ were used to generate polyclonal rabbit antibodies from a commercial source (ProSci Incorporated, USA).

### Nasopharyngeal infection model

HLA-expressing humanized mice (B6-DR4, B6-DQ8, B6-DR4/DQ8) have been previously described [12], [59], [60]. These mice were bred in a barrier facility at Western University and were routinely genotyped for the appropriate transgene(s). The mouse nasopharyngeal infection model has been described [61], with modifications. Briefly, mice were used at 9–13 weeks. Freshly grown exponential phase *S. pyogenes* cells (OD_600_ 0.2–0.3) were washed and suspended in Hanks balanced saline solution [HBSS; a total of ∼1×10^8^ (range 0.6–1.4×10^8^) CFU per 15 µl] and 7.5 µl was used to inoculate each nostril under Forane (isoflurane, USP) inhalation anesthetic (Baxter Corporation). Sham treated mice only received HBSS. Mice were sacrificed at the noted time points, and the cNTs, including the nasal associated lymphoid tissue, nasal turbinates, and maxillary sinuses were removed. Tissue was homogenized in HBSS, serially diluted, and plated on Trypticase Soy Agar with 5% Sheep Blood plates (Becton, Dickinson and Company, MD, USA), or used for cytokine analysis, or flow cytometry.

### *Ex vivo* experiments

For splenocyte activation experiments, cells were harvested from B6 or HLA-B6 mouse spleens, treated with ammonium-chloride-potassium (ACK) lysis buffer (15 mM NH_4_CL, 10 mM KHCO_3_, 0.1 mM EDTA) and suspended (2×10^5^ cells per well) in RPMI 1640 medium supplemented with 10% heat-inactivated fetal bovine serum (Sigma-Aldrich), 0.1 mm minimal essential medium (MEM) non-essential amino acids, 2 mm L-glutamine, 1 mm sodium pyruvate, 100 U ml^−1^ penicillin, 100 µg ml^−1^ streptomycin (all from Gibco Life Technologies) and 50 µM β-mercaptoethanol (Sigma). SAgs were added at the indicated concentrations and mouse IL-2 was determined after 18 h by enzyme-linked immunosorbent assay (eBioscience). Proliferation was measure by the addition of 1 µCi/well [^3^H]thymidine after 72 h and after another 18 h cells were harvested on fiberglass filters and [^3^H]thymidine incorporation was assessed on a 1450 Microbeta liquid scintillation counter (Wallac).

### Molecular modelling

The SpeA Tyr100→Ala mutation was predicted to disrupt the low-affinity MHC-II binding domain of SpeA based on a model of the crystal structure of SpeA in complex with HLA-DQ8. To generate this model, the structures of SpeA (PDB: 1FNU) [62] and HLA-DQ8 (PDB: 1JK8) [63] were superpositioned onto the known SEC3:HLA-DR1 complex (PDB: 1JWM) [64], and visualized using Pymol (pymol.sourceforge.net). Mutagenesis of *speA* was conducted using megaprimer-PCR (**Table S1**) to introduce the SpeA Tyr100→Ala mutation.

### Histology and immunofluorescence

Mice were fully anesthetized with Forane (isoflurane, USP) inhalation anaesthetic (Baxter Corporation) and perfused through the heart with sterile PBS containing heparin using a Gilson Minipuls 3 peristaltic pump (Middletown, WI, U.S.A) at a constant flow rate. Mice were then perfused with 10% neutral buffered formalin (BDH, VWR, West Chester, PA, USA) through the peristaltic pump. The head was soaked in 10 volumes of formalin for 24 hours and re-suspended in Shandon TBD-2 Decalcifier (TBD; Thermo Scientific, Kalamazoo, MI, USA) for 96 hours. TBD-decalcified heads were placed in formalin for 48 hours and washed with 1× PBS, and resuspended in 10-volumes of 1× PBS twice daily for 4 days, washed in 70% ethanol twice, and stored in 10-volumes of 70% ethanol. Cassettes were processed in Leica ASP300 fully enclosed paraffin wax tissue processor overnight using the ‘bone’ program and embedded in paraffin wax. Heads were serially sectioned between the first and second molar on a HM335E Microtome (Leica) into 5 micron sections, mounted on Fisherbrand Superfrost Plus microscope slides (Fisher Scientific, Fair Lawn, NJ, USA) and dried at 45°C for 48 h prior to storage/staining. Tissues were stained with H&E in a Leica Autostainer XL.

H&E stained slides were evaluated by an experienced mouse pathologist in a blinded fashion. The relative amount of mucus present covering the epithelia, the presence of red blood cells, and the presence of nucleated cellular debris on the surface of the epithelia was assessed and the presence and severity of these findings were used to assign a score of zero to two points to each of two sections per mouse (n = 5 mice per group). The scores were averaged to determine differences in histological pathology in the mice. Fluorescence staining was done with adjacent serial sections using a Goat α-*S. pyogenes* polyclonal (NB200-643; Novus Biologicals) at 1∶100 dilution, and donkey α-Goat Alexflour 595 (A-11058; Invitrogen) at 1∶1000 dilution. Images were captured using an upright BX61 fluorescent microscope (Olympus).

### Flow cytometry

For flow cytometry analysis, isolated cells (from cNTs, lymph nodes, or spleens) were aliquoted at 500,000 cells per 5 ml tube, and pre-treated with Fc block (hybridoma clone 2.4G2) prior to cell staining. Staining was done in panels using the following antibodies: α-CD3-APC (clone 145-2C11), α-CD3-PE Cy7 (clone 53–7.3), α-CD8 PE Cy7 (clone 53–6.7), α-CD45 PE (clone 30-F11), α-CD19 (clone MB19-1), α-NK1.1 (clone PK136), α-CD11c (clone N418), α-F4/80 (clone BM8), and α-GR1 (clone RB6-8C5) (all from eBioScience); α-CD4 APC Cy7 (clone GK1.5; Biolegend); and α-CD45 Alexafluor 700 (clone 30-F11; BD Biosciences). Dead cells were excluded using 7-AAD (BD Biosciences). Antibodies to stain cells for each panel were added, mixed, and incubated on ice in the dark for 30 minutes. Cells were washed twice with 1× PBS +5% FBS and resuspended in 500 µl of 1× PBS +5% FBS. Stained cells were run on a BD FACS Canto II flow cytometer (BD Biosciences). Standard compensations were used for each tissue using FACSdiva software.

### Multiplex cytokine analysis

Cytokine concentrations were determined from cNT homogenates isolated from mice treated with HBSS (sham), wild-type *S. pyogenes* MGAS8232, or isogenic *S. pyogenes* MGAS8232 ΔSAg strains at either 24 or 48 hours post-infection in HLA-B6 mice. Multiplex bead arrays were performed using the Mouse Cytokine 32-plex Discovery Array (Eve Technologies). Heat maps were generated using the Matrix2png algorithm [65] and data is shown as the average cytokine responses from 3–4 mice per group. Quantitative data from the cytokine analyses are shown in **Figure S2**.

### Vaccination experiments

For vaccination experiments, 6–8 week old HLA-B6 mice were injected subcutaneously with 1 µg of recombinant SpeA_Y100A_ (or sham) emulsified in Imject Alum Adjuvant (Thermo Fisher Scientific Inc.) every 2 weeks for a total of three injections. Two weeks following the last injection, mice were bled for antibody titers as determined by direct ELISA against 1 µg wild-type SpeA per well, and calculated as the reciprocal of the lowest serum dilution with readings 4-fold above background. Mice were challenged with ∼1×10^8^ CFU wild-type MGAS8232 as described above 24 hours after the final bleed and CFUs were determined 48 h post infection.

### Statistical analyses

When appropriate, individual data points, or the mean ± SEM, are shown, and *p* values were calculated using the Student's *t*-test with Prism software (GraphPad). A *p* value of less than 0.05 was determined to be statistically significant.

## Supporting Information

Figure S1 **Flow cytometry analysis of CD45^+^ leukocytes from the complete nasal turbinates (cNTs), lymph nodes, and spleens from sham,** ***S. pyogenes*** **MGAS8232, and** ***S. pyogenes*** **MGAS8232 ΔSAg infected HLA-mice at 48 h.** Data represents the mean ± SEM (n≥3 mice per group). Statistical significance is displayed as *p<0.05 by Student's *t*-test.
(PDF)

Figure S2 **Cytokine responses from the complete nasal turbinates (cNTs) from sham,** ***S. pyogenes*** **MGAS8232, and** ***S. pyogenes*** **MGAS8232 ΔSAg infected HLA-mice at 24 and 48 h post-infection.** (A) Pro-inflammatory or Th1-type cytokines, B) Th2-type cytokines, C) Chemokines, and D) Growth factors. Scale is shown in pg ml^−1^ (mean ± SEM) of total cNT homogenate (n≥3 mice per group). Statistical significance is displayed as *p<0.05 or **p<0.01 by Student's *t*-test.
(PDF)

Table S1 **Primers used in this study.**
(PDF)
