# Supplementary figures and images for: Bacterial Superantigens Promote Acute Nasopharyngeal Infection by Streptococcus pyogenes in a Human MHC Class II-Dependent Manner
Source: PLoS Pathog. 2014 May 29;10(5):e1004155. doi: 10.1371/journal.ppat.1004155 (PMC4038607; doi:10.1371/journal.ppat.1004155)

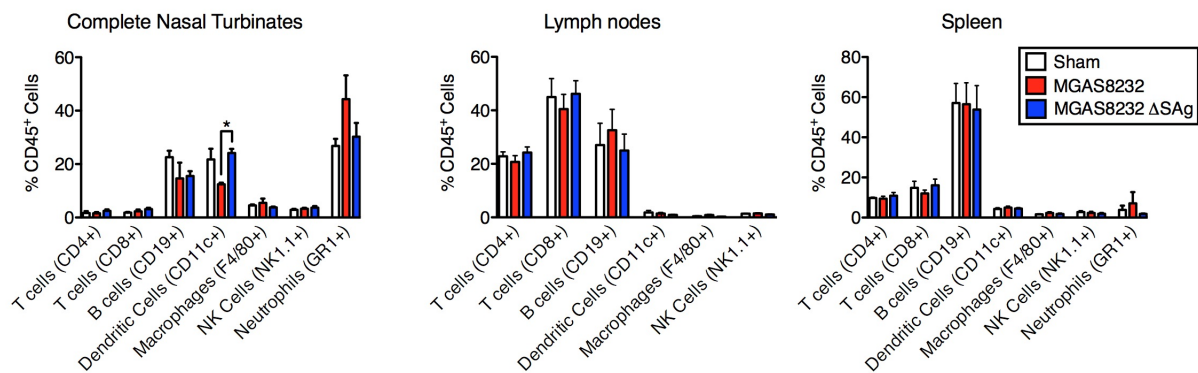

Kasper *et al.*, Figure S1

Supplement: Figure S1 — Flow cytometry analysis of CD45+ leukocytes from the complete nasal turbinates (cNTs), lymph nodes, and spleens from sham, S. pyogenes MGAS8232, and S. pyogenes MGAS8232 ΔSAg infected HLA-mice at 48 h. Data represents the mean ± SEM (n≥3 mice per group). Statistical significance is displayed as *p<0.05 by Student's t-test. (PDF) [file ppat.1004155.s001.pdf]

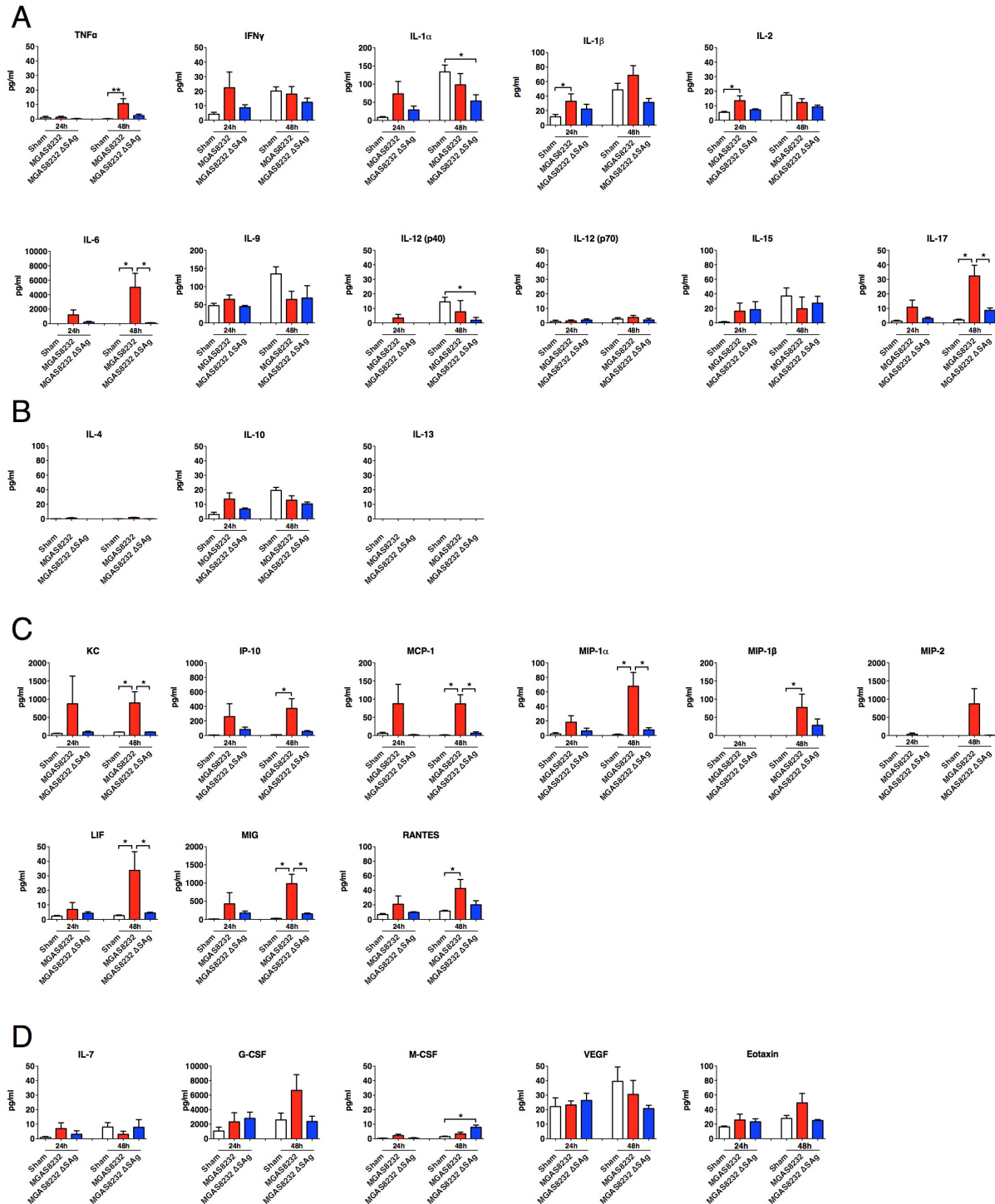

Kasper *et al.*, Figure S2

Supplement: Figure S2 — Cytokine responses from the complete nasal turbinates (cNTs) from sham, S. pyogenes MGAS8232, and S. pyogenes MGAS8232 ΔSAg infected HLA-mice at 24 and 48 h post-infection. (A) Pro-inflammatory or Th1-type cytokines, B) Th2-type cytokines, C) Chemokines, and D) Growth factors. Scale is shown in pg ml−1 (mean ± SEM) of total cNT homogenate (n≥3 mice per group). Statistical significance is displayed as *p<0.05 or **p<0.01 by Student's t-test. (PDF) [file ppat.1004155.s002.pdf]
