# Supplementary material for: Bacterial Superantigens Promote Acute Nasopharyngeal Infection by Streptococcus pyogenes in a Human MHC Class II-Dependent Manner
Source: PLoS Pathog. 2014 May 29;10(5):e1004155. doi: 10.1371/journal.ppat.1004155 (PMC4038607; doi:10.1371/journal.ppat.1004155)
Supplement: Table S1 — Primers used in this study. (PDF) [file ppat.1004155.s003.pdf]

Table S1. Primers used in this study.

Primer Name	Sequence (5'-3')[a]
**Primers for SAg expression constructs**	
NcoI-His-TEV-SpeA For	CCCCCATGGGCAGCAGCCATCATCATCATCATCACAGCAGCGGCGAAAACTTGTATTTCCAAGGCCAACAAGACCCCGATCCAAGCCA
BamHI-SpeA Rev	CCCGGATCCTTACTTGGTTGTTAGGTAGACTTCAAT
SpeA-Y100A For	AGTGCATGTATCGCCGGAGGGGTAACA
NcoI-His-TEV-SpeG For	CCCCCATGGGCAGCAGCCATCATCATCATCATCACAGCAGCGGCGAAAACTTGTATTTCCAAGGCGATGAAAATTTAAAAGATTTAAAA
BamHI-SpeG Rev	CCCGGATCCCTAGTACGTTTTTAAGTAGATATCAAA
NcoI-SpeL-Forward	GGGCCATGGGCAGCAGCCATCATCATCATCATCACAGCAGCGGCGAAAACTTGTATTTCCAAAGTGAAGAGACTATTAATATTAAGGAT
BamHI-SpeL-Reverse	GGGGGATCCTTATTTATTAAATTTAACTAAGTA
NcoI-SpeM-Forward	GGGCCATGGGCAGCAGCCATCATCATCATCATCACAGCAGCGGCGAAAACTTGTATTTCCAAAGTGATGCTGTGTTGGTTAATAGCGAA
BamHI-SpeM-Reverse	GGGGGATCCCTAATTTTTAGAAAAATCTTCGTT
SmeZ-TEV-KpnI For	CCCGGTACCGGTGGTGGCTCCGGTGAAAACTTGTATTTCCAAGGCTTAGAAGTAGATAATAATTCC
SmeZ-BamHI Rev	CCCGGATCCTTAGGAGTCAATTTCTATATC
**Primers for SAg gene chromosomal deletion constructs**	
BamHI-upstream speA MGAS8232 For	CCCGGATCCGCCAAGTTATGCCATTACTGTGTTG
PstI-upstream speA MGAS8232 Rev	CCCCTGCAGGAAGTCTACCTAACAACCAAGTAA
PstI-downstream speA MGAS8232 For	CCCCTGCAGCAATACTTTTTTATTGTTTTCCATTAATAT
KpnI-downstream speA MGAS8232 Rev	CCCGGTACCGCAAATGACAAATCGCTATATCAATAA
PstI-downstream speC MGAS8232 For	CCCCTGCAGTTTGATGATGTTAATCTTTTTCAT
KpnI-downstream speC MGAS8232Rev	CCCGGTACCCGGATATCAATTTTGTGGATTAAACG
BamHI-upstream speC MGAS8232 For	CCCGGATCCCGGTTACACCATGCGCACTGTCG
PstI-upstream speC MGAS8232 Rev	CCCCTGCAGTTCGATATTTATCTTGAAAAATAATTCATC
BamHI-upstream speG MGAS8232 For	CCCGGATCCGCCCAGCTTCAAGACGTTGGTTAA
PstI-upstream speG MGAS8232 Rev	CCCCTGCAGAAATTCTAAAATGAACTTAGCCAC
PstI-downstream speG MGAS8232 For	CCCCTGCAGGATATCTACTTAAAAACGCACTAG
KpnI-downstream speG MGAS8232 Rev	CCCGGTACCGGAAGATCAAGCCAACCCAAGAAA
BamHI-upstream speL/M MGAS8232 For	CCCGGATCCCCTATGGACGTAGACAAATATGTTG
PstI-upstream speL/M MGAS8232 Rev	CCCCTGCAGTTATTCACAAACAAAGAAAATTAATTAGTA
PstI-downstream speL/M MGAS8232 For	CCCCTGCAGCAAAGTCAAGGTATTTTTTTTCAT
KpnI-downstream speL/M MGAS8232 Rev	CGCGGTACCCGGTGTATTGACATTGATGTATTC
BamHI-upstream smeZ MGAS8232 For	GGGGGATCCGGGGAATTATGCCAATTGTCTCTA
PstI-upstream smeZ MGAS8232 Rev	CCCCTGCAGAAAAATAAGTTTTGTTTTTTTCATAAATAG
PstI-downstream smeZ MGAS8232 For	CCCCTGCAGTTAGATATAGAAATTGACTCCTAATTC
KpnI-downstream smeZ MGAS8232 Rev	GGGGGTACCGGGCAATTGTTTAACTGGTTAATTAG

Primers for the SpeA and SpeA$_{Y100A}$ complementation constructs

KpnI-tsf For	GGGGGGGGTACCACTTGCTCAATTGAACCACG
EcoRI-MCS-tsf Rev	TTTTTTGAATTCGATATCAAGCTTATCGATACCGTCGACCTCGAGGGGGGGCCCTCCGTTTGACACAACAAAAAGA
EcoRI-MCS-pepO For	GGGGGGGAATTCCTGCAGCCCGGGGGATCCACTAGTTCTAGACACCAATAAGGAAGCAAAAA
NotI-pepO Rev	GGGGGGGCGGCCGCAGCCTAAATGATTGGTGGA
SpeI-speA Comp For	GGGGGGACTAGTATTTTATAATAAAATTATTAATATAAGTTAA
SalI-speA Comp Rev	GGGGGGGTCGACAAAACCGCTCATCAAATGA

Screening and sequencing primers for genomic integrations

Int/Screen speA For	GCAAGCTACGGTTACGAAATTG
speA Screen II 8232 Rev	CCATCCTTTTTTGATTCTCC
speC Int II For	GCAAAGCACTGGTTCGATGT
speC Int II Rev	GGATAACCTTAACCGCGCTAC
speG int II For	GCCCTCGTCAGAATGACTGT
speG Screen II Rev	CCGATACCGATCACCACAAG
speL/M Int II For	GGCTTGAGTTATGTGTCTTTA
speL/M Screen II Rev	GTTTGGTGGTATAGATGTAGCAAGG
smeZ Int II For	CATGCCTGCTCAAACAAGATT
smeZ Screen II Rev	ATACGACTCCATCTCATTATAGC

Superantigen gene internal screening primers.

speA RT For	AAAGTTGCCATCTCTTGGTTC
speA RT Rev	CAAGAGGTATTTGCTCAACAAGAC
speC RT For	TTTGAGCAGGCGTAATTCCT
speC RT Rev	TTCAACGACACACACATTAAACA
speG RT For	ACCCCATGCGATTATGAAAA
speG RT Rev	GGGAGACCAAAAACATCGAC
speL RT For	ATAAGTCAGCACCTTCCTCTTTC
speL RT Rev	AAATCTCCCGTTACCTTCCA
speM RT For	AACTTCTTCTTCCTTAAAGCGTCT
speM RT Rev	TGCTGTGTTGGTTAATAGCGA
smeZ RT For	TTTCTCGTCCTGTGATTGGA
smeZ RT Rev	AATGGGACGGAGAACATAGC

Mouse genotyping primers

DQA (1027) For	GAAGACATTGTGGCTGACCATGTTGCC
DQA (1029) Rev	AGCACAGCGATGTTTGTCAGTGCAAATTGCGG
DQ8b (vA) For1	AGGATTTGGTGTACCAGTTTAAGGGCAT
DQ8b (vT) For 2	AGGATTTGGTGTTCCAGTTTAAGGGCAT

DQ8b (vii) Rev	TGCAAGGTCGTGCGGAGCTCCAA
DR4A (1101) For	GGAGATAGTGGAACTTGCGG
DR4A (1104) Rev	CCGATCACCAATGTACCTCC
DR4B (1098) For	GTTTCTTGGAGCAGGTTAAACA
DR4B (1099) Rev	CTGCACTGTGAAGCTCTCAC

[a] Underlined sequences in primers indicate restriction endonuclease sites used for cloning purposes.
